# Abnormalities of hair structure and skin histology derived from CRISPR/Cas9-based knockout of phospholipase C-delta 1 in mice

**DOI:** 10.1186/s12967-018-1512-9

**Published:** 2018-05-25

**Authors:** Yu-Min Liu, Wei Liu, Jun-Shuang Jia, Bang-Zhu Chen, Heng-Wei Chen, Yu Liu, Ya-Nan Bie, Peng Gu, Yan Sun, Dong Xiao, Wei-Wang Gu

**Affiliations:** 1 Institute of Comparative Medicine & Laboratory Animal Center, Southern Medical University, Guangzhou, 510515 China; 2 Songshan Lake Pearl Laboratory Animal Sci. & Tech. Co., Ltd., Dongguan, 523808 China; 3 Guangdong Provincial Key Laboratory of Cancer Immunotherapy Research and Guangzhou Key Laboratory of Tumor Immunology Research, Cancer Research Institute, Southern Medical University, Guangzhou, 510515 China; 4 Zhongshan School of Medicine, Sun Yat-sen University, Guangzhou, 510080 China; 5 Jing Brand Co., Ltd., Daye, 435100 Hubei China

**Keywords:** Phospholipase C-delta 1 (PLCD1), Nude mice, Tibet minipigs, CRISPR/Cas9 technology, Hairless

## Abstract

**Background:**

Hairless mice have been widely applied in skin-related researches, while hairless pigs will be an ideal model for skin-related study and other biomedical researches because of the similarity of skin structure with humans. The previous study revealed that hairlessness phenotype in nude mice is caused by insufficient expression of phospholipase C-delta 1 (PLCD1), an essential molecule downstream of Foxn1, which encouraged us to generate PLCD1-deficient pigs. In this study, we plan to firstly produce PLCD1 knockout (KO) mice by CRISPR/Cas9 technology, which will lay a solid foundation for the generation of hairless PLCD1 KO pigs.

**Methods:**

Generation of PLCD1 sgRNAs and Cas 9 mRNA was performed as described (Shao in Nat Protoc 9:2493–2512, 2014). PLCD1-modified mice (F0) were generated via co-microinjection of PLCD1-sgRNA and Cas9 mRNA into the cytoplasm of C57BL/6J zygotes. Homozygous PLCD1-deficient mice (F1) were obtained by intercrossing of F0 mice with the similar mutation.

**Results:**

PLCD1-modified mice (F0) showed progressive hair loss after birth and the genotype of CRISPR/Cas9-induced mutations in exon 2 of PLCD1 locus, suggesting the sgRNA is effective to cause mutations that lead to hair growth defect. Homozygous PLCD1-deficient mice (F1) displayed baldness in abdomen and hair sparse in dorsa. Histological abnormalities of the reduced number of hair follicles, irregularly arranged and curved hair follicles, epidermal hyperplasia and disturbed differentiation of epidermis were observed in the PLCD1-deficient mice. Moreover, the expression level of PLCD1 was significantly decreased, while the expression levels of other genes (i.e., Krt1, Krt5, Krt13, loricrin and involucrin) involved in the differentiation of hair follicle were remarkerably increased in skin tissues of PLCD1-deficient mice.

**Conclusions:**

In conclusion, we achieve PLCD1 KO mice by CRISPR/Cas9 technology, which provide a new animal model for hair development research, although homozygotes don’t display completely hairless phenotype as expected.

**Electronic supplementary material:**

The online version of this article (10.1186/s12967-018-1512-9) contains supplementary material, which is available to authorized users.

## Background

Currently, the minipigs are becoming the most widely used large laboratory animals due to their short stature, easy operating, and sharing anatomical, physiological and biochemical similarities to humans. On account of observable similarities between porcine and human skin structure, pigs are considered as a perfect animal model for skin-related studies, such as skin grafting [2], cosmetic identification [3], ultraviolet radiation [4], skin cancer [5], burns [6], frostbite [7] and etc. However, because of pig skin coated with shaggy hairs, shaving hair process is inevitable before skin test or surgery. Hence, it makes sense to generate hairless pigs to eliminate the need for hair removal procedures before experiment and surgery, and avoid the skin damage caused thereby.

So far, some hairless animal models, including nude mice, SKH hairless mice, hairless guinea pigs and Yucatan miniature pig, have been widely applied in skin-related studies (i.e., hair tonic effect, skin allergies, skin grafting treatment and ultraviolet radiation response, etc.) [8–10]. Presently, China has nurtured many miniature pig strains, including Wuzhishan miniature pigs, Guizhou miniature pigs, Bama miniature pigs, Banna miniature pigs and Tibetan minipigs, etc. [11–14]. However, Chinese scientists has not yet nurtured hairless miniature pig strains, which are urgently required for biomedical researches. It was reported that transgenic mice expressing DKK1 transgene under control of a human K14 promoter showed hairless phenotype [15]. In our previous study, we generated transgenic cloned pigs that expressed pDKK1 transgene under control of K14 promoter, but none of DKK1 transgenic pigs displayed hairless phenotype as expected [16].

Nude mice exhibit hairless and congenital athymia by a loss-of-function mutation in the transcription factor Foxn1 gene [17, 18]. In the hair shaft and IRS of nude mice, impaired keratinization and structural defects were found, causing the formation of shorter, broken hair shafts that seldom penetrate the skin surface [19]. The previous study revealed that phospholipase C-delta 1 (PLCD1/PLCδ1) is an essential molecule downstream of Foxn1 in normal hair development, and strongly suggests that hairlessness phenotype in nude mice is caused by insufficient PLCD1 expression [20, 21]. Like nude mice, PLCD1 knockout (KO) mice generated by homologous recombination displayed hairless phenotype, but there are normally functional thymus in PLCD1 KO mice [20, 21].

The CRISPR/Cas9 system is an immune defense system found in bacteria and archaea that used to resist viral invasion, consists of RNAs that specifically recognize the target DNA sequence and the Cas9 endonuclease [22]. Since 2013, CRISPR/Cas9 gene editing technology has been widely used in many fields [23–25]. The generation of genetic modified animals by using CRISPR/Cas9 gene editing technology has simple experiment procedure, short experiment cycle and low cost, compared with engineered nucleases technology (TALENs and ZFN) [26, 27]. Against this background, we will intend to generate PLCD1 knockout Tibetan miniature pigs via CRISPR/Cas9 technology, and finally achieve hairless minipigs. At first, we want to produce PLCD1 KO mice by CRISPR/Cas9 technology, which will lay a solid foundation for the generation of PLCD1 KO pigs with hairlessness phenotype.

## Methods

### Animals

All animals used in this study were maintained in standard cages in an assessment pathogen–free (SPF) animal facility on a daily 12-h light/dark cycle. All animal protocols were approved by the Institutional Animal Care and Use Committee (IACUC) at the Institute of Laboratory Animal Center, Southern Medical University (L2016090).

### Generation of Cas 9 mRNA and PLCD1 sgRNA

The proper target sequence of PLCD1 sgRNAs was acquired from Optimized CRISPR Design (http://crispr.mit.edu). Generation of PLCD1 sgRNAs and Cas 9 mRNA was performed as described [28]. pUC57-sgRNA expression vector was purchased from Addgene (Plasmid 51132). Complimentary oligonucleotides containing the PLCD1 sgRNA target sequences were annealed and cloned into the *Bsa* I site of pUC57-sgRNA. These recombination plasmids were then sequenced to screen correct insertion of the target sequences. pUC57-sgRNA-PLCD1 linearized by *Dra* I was purified, followed by in vitro transcription using MEGAshortscript™ Kit (Ambion). Cas9 mRNA was synthesized using the mMessagemMachine T7 Ultra Kit (Ambion), followed by purification. The quality of Cas9 mRNA and sgRNAs was confirmed by agarose gel electrophoresis.

### Generation of PLCD1 konckout mice and breeding

The C57BL/6J mice (3–4 weeks old), supplied by Laboratory Animal Center, Southern Medical University, were used as the source of embryos for the micromanipulation and for the subsequent breeding trials. A mixture of in vitro transcribed RNA (Cas9 mRNA,100 ng/μl; sgRNA, 50 ng/μl) was injected into the cytoplasm of one cell-stage fertilized embryos. Zygotes that survived were transferred into the oviducts of pseudopregnant foster mothers. The heterozygous mutant mice with similar mutation were intercrossed to produce the homozygous mutant mice.

### Genotyping

Genomic DNA from mouse tail biopsies was prepared according to the protocol of genomic DNA extraction kit (Tiangen). The sequences of the forward primer (FP) and reverse primer (RP) used to identify the genetically modified mice were: PLCD1-F: 5′-AGACGTCTTGCCTGTGAAGG-3′ and PLCD1-R: 5′-CGCTCTGATCCACCCATTGT-3′. PCR reaction conditions were as follows: pre-denaturation at 95 °C for 5 min, followed by 30 amplification cycles of denaturation at 95 °C for 30 s, primer annealing at 60 °C for 50 s, and extension at 72 °C for 60 s, and finally an additional extension at 72 °C for 10 min. The amplified PCR products were 675 bp in length. PCR products were purified with gel extraction purification kit, and then cloned into the pMD-19 T vector (Takara) following the manufacturer’s instructions. The recombinant colonies selected on LB/IPTG/X-Gal plates were screened by PCR using PLCD1 gene-specific primers mentioned above, and then Sanger sequencing was applied to detect mutations. At least 10 clones were sequenced from each mouse.

### RNA extraction and quantitative real-time PCR (qRT-PCR)

To detect the mRNA levels of PLCD1, PLCB1, PLCG1, PLCE1, Krt1, Krt5, Krt13, loricrin and involucrin, total RNA was isolated from skin tissue samples (both abdomen and back) using Trizol Reagent (Takara). cDNA was synthesized using the PrimeScript RT reagent Kit (Takara). The primers for qRT-PCR were showed in Additional file 1: Table S3.

### Histological analysis

Skin tissue samples were taken from the same site on back of mutant mice and wild type mice (6 weeks old) after cervical dislocation, fixed with 4% paraformaldehyde (PFA) in PBS, and embedded in paraffin as described previously [29–33]. Four millimeter thick sections were mounted on slides and stained with hematoxylin and eosin (H&E staining) according to standard procedures. The immunohistochemical (IHC) staining of PLCD1, Ki67 and Krt13 followed the standard streptavidin-peroxidase (SP) protocol.

### Off-target analysis

Off-target cleavage sites were predicted and searched. In brief, exonic sites with over 15 bp that matched to the 20 bp sequence of sgRNA and NGG (PAM, 3 bp) in the mouse genome were predicted as OTS. All of the potential OTSs were amplified through PCR, and the PCR products were sequenced to confirm whether off-targets exist. Additional file 1: Tables S4 and S5 showed the sequences of OTSs and primer pairs used to amplify the candidate OTSs.

### Statistical analysis

Data were presented as mean ± SD. Statistical analysis was performed using a SPSS 13.0 software package and Graphpad 5.0 software. Independent-Sample T test was used for comparisons of 2 independent groups. Statistical significance was assessed by the Student’s *t* test (**P < 0.01; ^#^*P* < 0.001).

## Results

### Design of CRISPR/Cas9 system

**Fig. 1:**
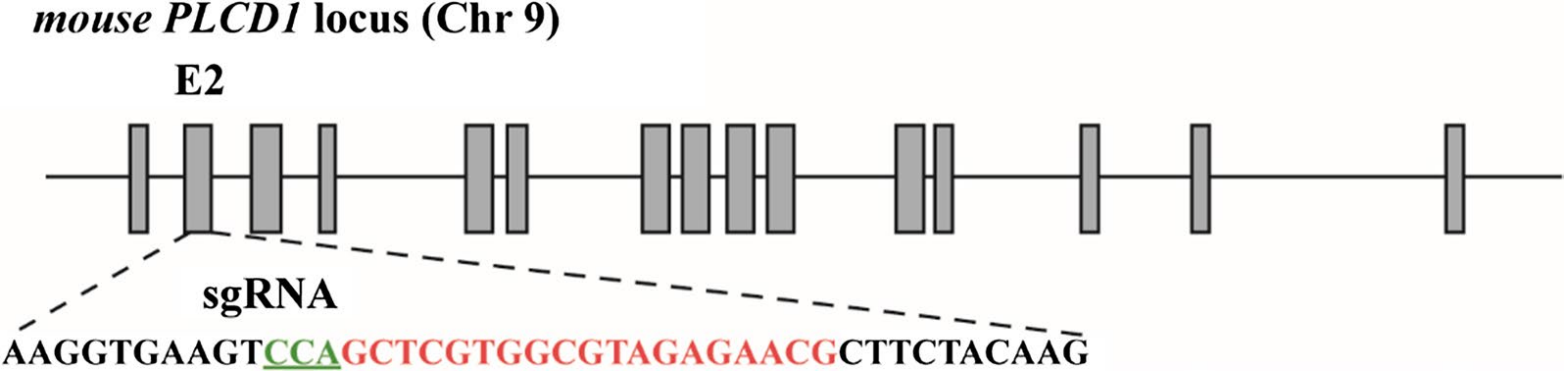
Mouse PLCD1 gene structure and sgRNA design. Diagram of mouse PLCD1 locus, showing 11 exons. sgRNA guiding sequence and PAM at the target site are highlighted in red and green, respectively

It has reported that PLCD1 is required for skin stem cell lineage commitment and PLCD1 knockout mice with disruption of X and Y domains (corresponding to exons 7 and 8) by homologous recombination in mouse ES cells undergo progressive hair loss in the first postnatal hair cycle [34–36]. To generate PLCD1-modified mice by CRISPR/Cas9 technology, exons 2 (beginning of the translation PLCD1), 7 and 10 (corresponding to X and Y domains, respectively) of PLCD1 were scanned for potential sgRNA target sequences using Optimized CRISPR Design (http://crispr.mit.edu). Three sgRNAs were designed (Additional file 1: Table S1), and then the efficiency was estimated by SSA (Single-strand annealing) assay in vitro, as described previously [23]. Finally, sgRNA targeting exon 2 was chose to modify PLCD1 (Fig. 1).

### Generation of PLCD1-modified mice

**Fig. 2:**
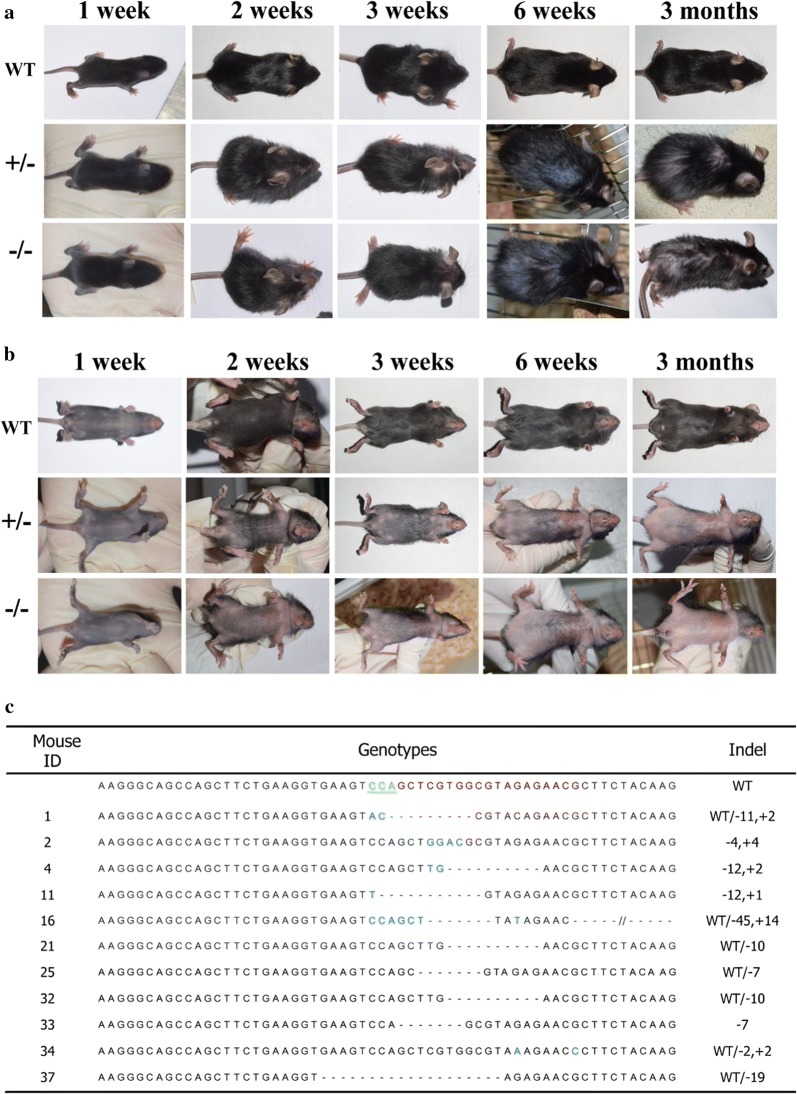
CRISPR/Cas9-mediated modification of the PLCD1 gene in founder mice. **a**, **b** Dorsal (**a**) and ventral (**b**) views of wild-type (WT), monoallelic (±) and biallelic (−/−) PLCD1-modified mice. **c** The sequencing-based genotype analysis of founder mice

PLCD1-modified mice were generated via co-microinjection of PLCD1-sgRNA (targeting exon 2) and Cas9 mRNA into the cytoplasm of C57BL/6J zygotes. Three injection sessions yielded 28 pups, 26 of which survived until weaning (Additional file 1: Table S2). Among these survived pups, 24 pups began to shed hair at 2 weeks old, and showed bald abdomen and sparse hair on back and head at 3 weeks old (Fig. 2a, b). At 3 months, a variety of hair loss were observed, 5 (#2, #4, #11, #21 and #25) out of 24 mice remained nearly hairless on abdomen, and sparse hair on back and head (~ 19%) (Fig. 2a, b). The rest of two mice (#23 and #38) displayed normal hair.

All mice (n = 26) were genotyped by sequencing for CRISPR/Cas9-induced mutations in exon 2 of the PLCD1 locus. Amplified DNA fragments by PCR using one primer pair that flanks the sgRNA target sequence were subcloned, and subsequently sequenced. The mutations around the target site were identified in 11 of 26 mice (~ 42.3%), and four mice (~ 15.4%) were bi-allelic mutations (Fig. 2c).

### Generation of PLCD1-deficient mice (F1)

**Fig. 3:**
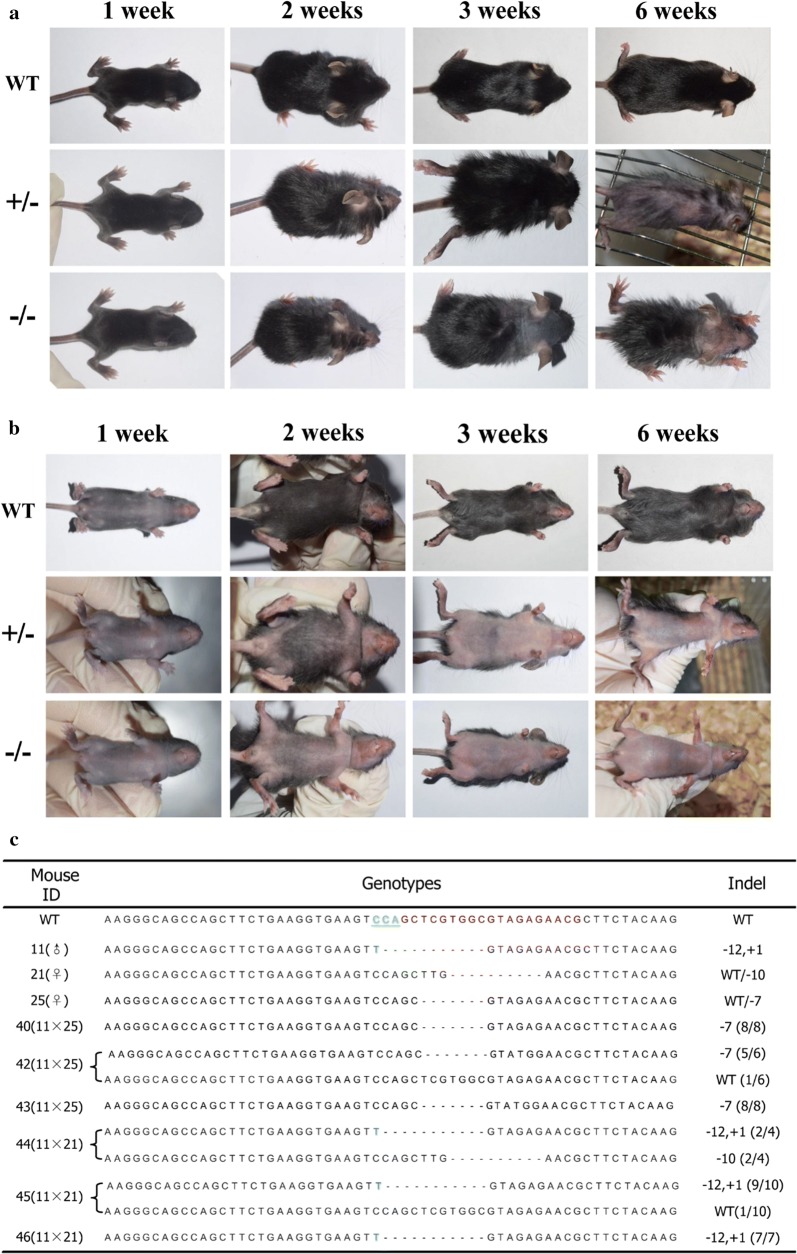
The generation of PLCD1-deficient mice (F1). **a**, **b** Dorsal (**a**) and ventral (**b**) views of wild-type (WT), heterozygous (±) and homozygous (−/−) PLCD1-deficient mice. **c** The sequencing-based genotype analysis of PLCD1-deficient mice (F1)

To obtain mutant mice homozygous(−/−) for PLCD1 mutation, the conventional method is mating PLCD1-modified mice (F0) with wild-type mice to obtain mutant mice heterozygous (∓) for PLCD1 mutation, and then PLCD1 heterozygous mutant mice were intercrossed to produce the homozygous PLCD1-deficient mice. Thus, it is clear that the conventional method is time-consuming. In this study, PLCD1-modified mice (F0) (#11, #21 and #25) with similar mutation were intercrossed to produce PLCD1 homozygous mutant mice (F1). Finally, we obtained 7 PLCD1-deficient mice (F1), among which 4 PLCD1-deficient mice (#40, #41, #42 and #43) were produced by breeding mouse #11 with mouse #25, and three PLCD1-deficient mice (#44, #45 and #46) were generated by breeding mouse #11 with mouse #21. 6 out of 7 mutant mice began to shed hair at 2 weeks old which was observed in PLCD1-modified mice (F0), and demonstrated bald abdomen and sparse hair on back at 3 weeks old which is more obvious than that of PLCD1-modified mice (F0). Furthermore, the PL CD1-deficient mice (F1) showed bald head and neck at the age of 3 weeks, which was not observed in PLCD1-modified mice (F0). The distribution of abdominal and dorsal hair remained the same stage at the age of 6 weeks. The hair of PLCD1-deficient mice (F1) was softer and thinner than that of wild-type mice and PLCD1-modified mice (Fig. 3a, b).

All of PLCD1-deficient mice (F1) (n = 7) were genotyped by sequencing for CRISPR/Cas9-induced mutations as mentioned above. The anticipated mutations were identified in 6 out of 7 mice (Fig. 3c). Among 6 PLCD1 mutant mice (F1), there are three homozygous PLCD1-deficient mice (F1), including 2 mice (#40 and #43) with deletion of 7 bp and one mouse with deletion of 11 bp and insertion of 1 bp. In summary, homozygous PLCD1-deficient mice were obtained in first-filial generation, indicating that intercrossing of genetically modified mice (founder; F0) produced by CRISPR/Cas9 technology to generate homozygote is feasible and time-saving.

### The histological analysis in the skin of PLCD1-deficient mice

**Fig. 4:**
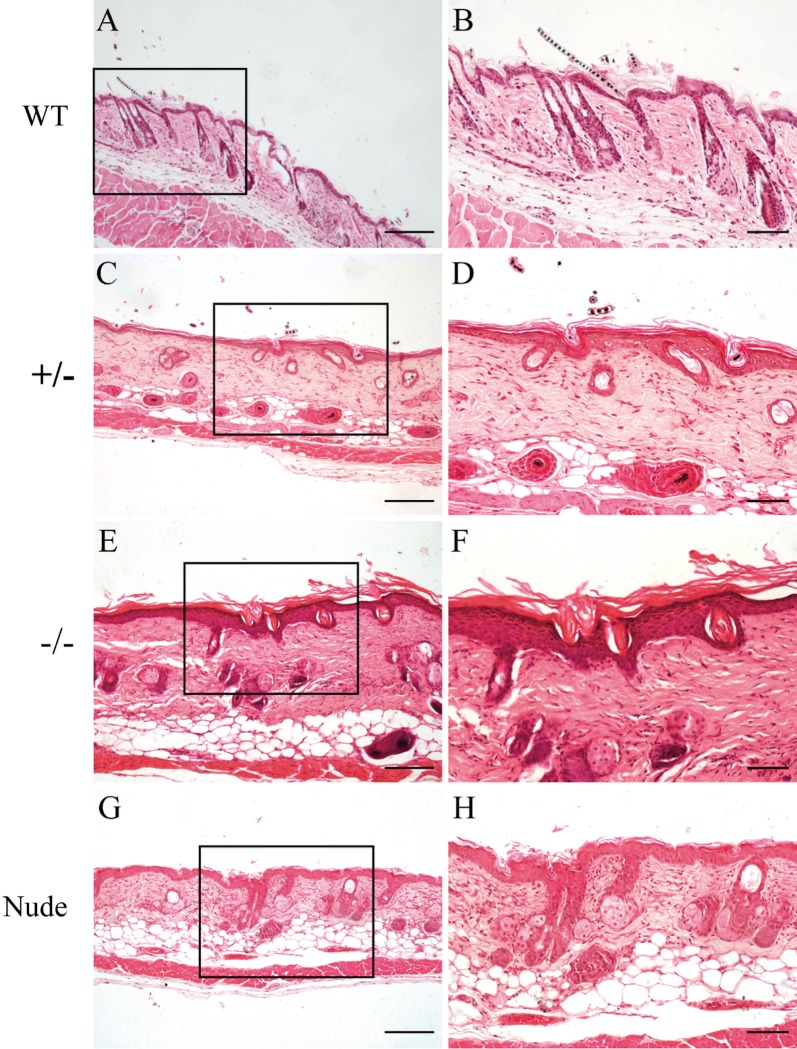
Histological abnormalities in the skin of founder mice. H&E staining of dorsal skin sections from wild-type (**A**, **B**), monoallelic (±) (**C**, **D**) and biallelic (−/−) (**E**, **F**) PLCD1-deficient mice, and nude mice (**G**, **H**). In **B**, **D**, **F** and **H** are higher magnifications of the black boxes indicated in **A**, **C**, **E** and **G**, respectively. Scale bar: **A**, **C**, **E**, **G**: 100 μm; **B**, **D**, **F**, **H**: 50 μm

**Fig. 5:**
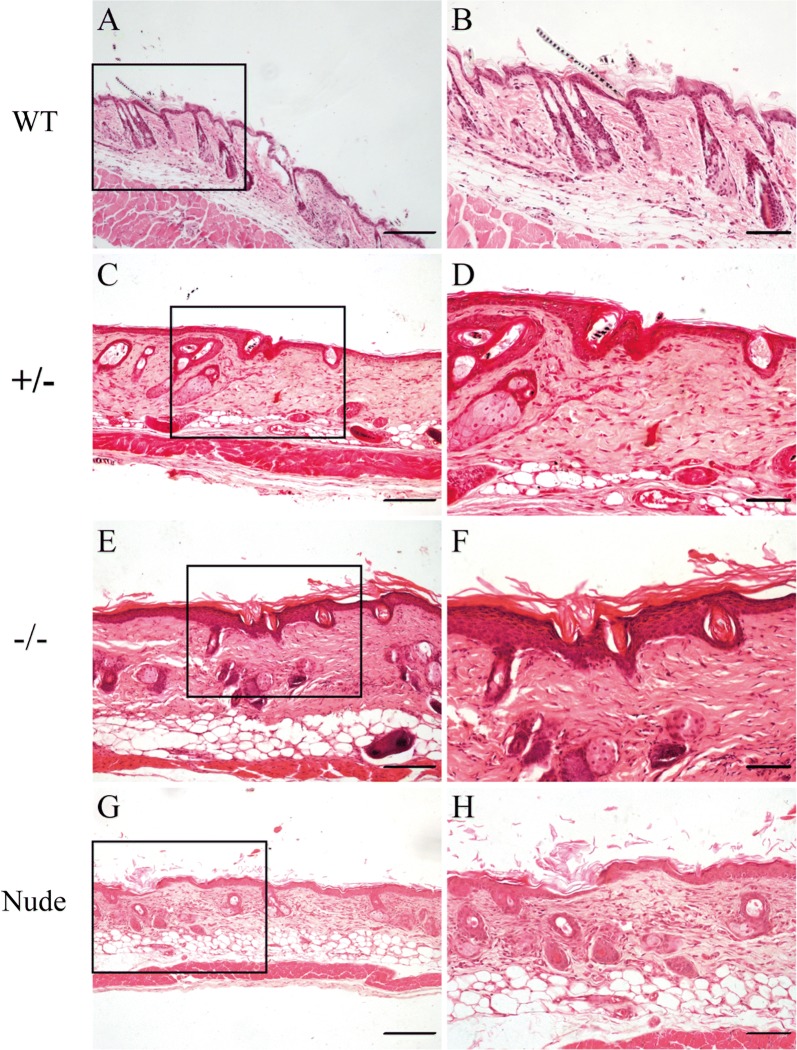
Histological abnormalities in the skin of PLCD1-deficient mice (F1). H&E staining of dorsal skin sections from wild-type (**A**, **B**), heterozygous (±) (**C**, **D**) and homozygous (−/−) (**E**–**F**) PLCD1-deficient mice, and nude mice (**G**, **H**). In **B**, **D**, **F** and **H** are higher magnifications of the black boxes indicated in **A**, **C**, **E** and **G**, respectively. Scale bar: Fig. 4**A**, **C**, **E**, **G**: 100 μm; Fig. 4**B**, **D**, **F**, **H**: 50 μm

Almost all PLCD1-modified mice displayed the defect in the fur development, whereas control mice had well-developed coats (Figs. 2a, b and 3a, b), which prompted us to investigate skin histological structure of PLCD1-deficient mice (Figs. 4 and 5). We found that the number of hair shaft decreased in the skin of PLCD1-deficient mice (Figs. 4C–F and 5C–F), compared with that of wild-type mice (Figs. 4A, B and 5A, B). Additionally, most of hair shaft in PLCD1-deficient mice are abnormal and fail to penetrate the epidermis (Figs. 4C–F and 5C–F), whereas hair shaft in wild-type mice penetrate the epidermis (Figs. 4A, B and 5A, B). In PLCD1-deficient mice, some hair canals were occluded by differentiated keratinocytes (Figs. 4D, F and 5D, F). Moreover, interfollicular epidermis (IFE) of PLCD1-deficient mice (Figs. 4C–F and 5C–F) was thicker than that of wild-type mice. Together, these findings demonstrate that PLCD1-deficient mice display abnormal histology, in the skin which is almost similar to those of nude mice.

### PLCD1 and other related gene expression in the skin of PLCD1-deficient mice

**Fig. 6:**
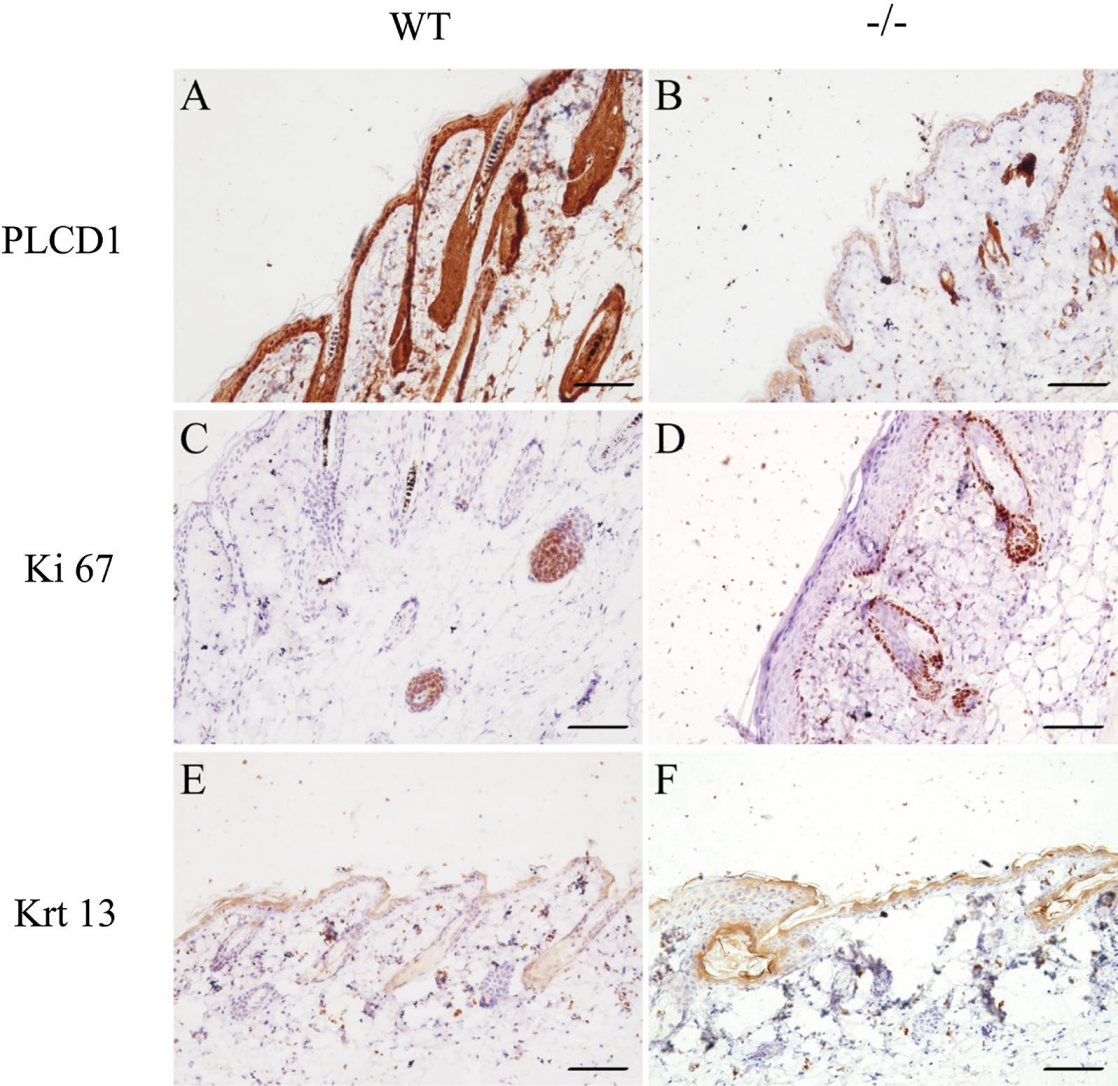
The immunohistochemical (IHC) staining of PLCD1, Ki67 and Krt13 in the skin of PLCD1-deficient mice. IHC staining with anti-PLCD1 antibody (**A**, **B**), anti-Ki67 antibody (**C**, **D**) and anti-Krt 13 antibody (**E**, **F**) of dorsal skin sections from wild-type and PLCD1-deficient (−/−) mice. Scale bar: 50 μm

**Fig. 7:**
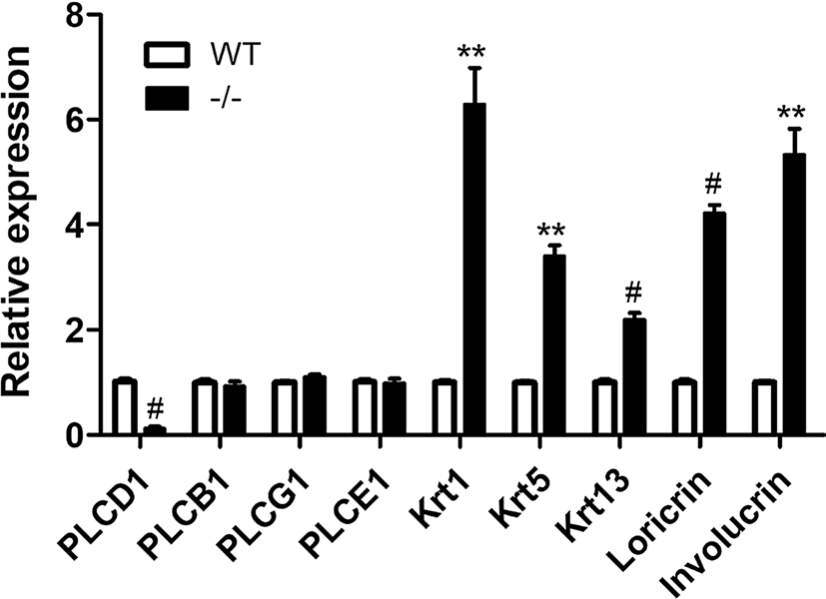
qRT-PCR analysis of the expression of PLCD1 and other related genes in the skin of PLCD1-deficient mice

Because abnormal epidermal and hair follicle morphologies were observed in skin from PLCD1-deficient mice, we investigated how the lack of PLCD1 influences the cell proliferation and differentiation in these structures. Thus, the expression levels of PLCD1 and other members of PLC family/delta family (i.e., PLCB1, PLCG1 and PLCE1), and these genes (i.e. Ki67, Krt1, Krt5, Krt13, loricrin and involucrin) involved in the growth and differentiation of hair follicle and epithelial tissues [37–41] were examined in the skin tissues of PLCD1-deficient mice by IHC (Fig. 6) or qRT-PCR (Fig. 7). The expression level of PLCD1 was very significantly decreased (Figs. 6A, B and 7), suggesting that CRISPR/Cas9-induced mutation in exon 2 of PLCD1 locus results in PLCD1 deficiency in homozygous PLCD1-deficient mice. There were no significant differences in the expression levels of PLCB1, PLCG1 and PLCE1 between wild-type mice and PLCD1-deficient mice (Fig. 7). More importantly, the expression levels of Krt1, Krt5, Krt13, loricrin and involucrin involved in the differentiation of hair follicle and epithelial tissues were remarkerably increased in skin tissues of PLCD1-deficient mice (Figs. 6E, F and 7), while the number of hyperproliferative Ki67-positive cells in skin tissues of PLCD1-deficient mice were significantly increased compared with control (Fig. 6C, D). Summarily, our results reveal the epidermal hyperplasia and disturbed differentiation of epidermis in PLCD1-deficient mice.

### Off-target analysis of PLCD1-deficient mice

Off-target effect is a major drawback concern of the CRISPR/Cas9 system [42, 43]. To examine whether off-target occurred in these genetically modified mice, possible off-target sequences within mouse genome were predicted using the CRISPR Design Tool (http://crispr.genome-engineering.org). The sgRNA targeting PLCD1 can potentially recognize 40 putative off-target sequences that have variable numbers of base mismatches. Only three of these are exonic sites (Additional file 1: Table S4). Approximately 500–800 bp genomic fragments containing off-target site were amplified by PCR using the primers listed in Additional file 1: Table S5, and subject to sequencing analysis. As a result, none of the sequencing reads exhibited any mutations, suggesting that no off-target occurred in any of 6 PLCD1-deficient mice (F1).

## Discussion

As mentioned in “Background“, hairless mice (i.e., SKH hairless mice) have been widely applied in skin-related studies [9]. Hairless pigs will be an ideal model for skin-related study and other biomedical researches. Currently, Yucatan miniature pig, a world’s only hairless pig strain, has been used in skin studies [10]. The significant applications of hairless pigs encourages us to generate hairless miniature pig strains based on miniature pig strains nurtured in China. In our previous study, we have produced transgenic cloned pigs expressing pDKK1 transgene under control of K14 promoter by using somatic cell nuclear transfer (SCNT), however unfortunately, DKK1 transgenic pigs didn’t display hairless phenotype as expected [16]. The absence of hairless phenotype in pDKK1 transgenic pigs may be due to the following potential reasons: (1) the expression level of pDKK1 transgene is not high enough to cause hairless phenotype, (2) the observing time is not enough and (3) DKK1 plays a different role in pigs [16]. Thus, we decided to look for other candidate gene to generate hairless pigs.

Nude mice exhibit hairless and congenital athymia by a loss-of-function mutation in the transcription factor Foxn1 gene [17, 18]. The previous study revealed that PLCD1, which is a key molecule in the phosphoinositide signaling pathway and is required for skin stem cell lineage commitment, is an essential molecule downstream of Foxn1 in normal hair formation, and strongly suggests that hairlessness in nude mice is caused by insufficient expression of PLCD1 [20, 21]. The PLCD1-deficient mice generated by homologous recombination showed hair abnormalities similar to nude mice, such as a lack of certain hair keratins and the twist hair shafts. In addition, PLCD1 expression was remarkably decreased in the skin of nude mice [34]. Therefore, we intend to establish PLCD1 KO Tibetan miniature pigs through the CRISPR/Cas9 gene editing technology to produce hairless pigs which will provide a more appropriate animal model for skin-related studies, cosmetics and drug testing. In this study, before establishing PLCD1 KO pigs, we want to firstly generate PLCD1 KO mice by CRISPR/Cas9 gene editing technology, and then determine whether these PLCD1-deficient mice can exhibit hairless phenotype. In the present study, we have successfully generated CRISPR/Cas9-mediated PLCD1 deficiency in mice, most of which showed bald abdomen and sparse hair on back and head rather than nude phenotype over the whole body. The absence of complete hairless phenotype in PLCD1-deficient mice may be due to the following possible reasons: (1) the target locus of sgRNA is not significant enough to induce the complete loss of PLCD1 function, and (2) the deletion with a small snippet in PLCD1 locus does not result in the dysfunction of PLCD1 protein.

Phosphoinositide metabolism, an important intracellular signaling system, is involved in various cell functions, covering secretion of hormones, transduction of neurotransmitters, growth factor signaling, membrane trafficking and regulation of the cytoskeleton [44]. Phospholipase C (PLC) is one of the key enzymes in this system [45]. PLCD1, δ-type PLC isozyme, is considered to be the most basic isoform among PLC family members because its structure is the simplest, comprising a PH domain [46]. The previous studies have revealed that PLCD1 is essential for normal hair formation, and PLCD1 KO mice show marked hair loss which is similar to that of nude mice [20, 34]. Furthermore, PLCD1 KO mice displayed symptoms of skin inflammation [47]. PLCD1 and PLCD3 have synergistic effects on the murine hair follicle in specific regions of the body surface [36, 48]. Although other studies have revealed the mutations detected in PLCD1 are involved in the development of leukonychia [49] and cancers, such as breast cancer [50], pancreatic cancer [51], and gastric cancer [52] and so on, the PLCD1-deficient mice produced in this study showed progressive hair loss without other detected abnormalities.

## Conclusion

In conclusion, we achieve PLCD1 KO mice by CRISPR/Cas9 technology, which may provide a model for exploring the functions of PLCD1 signaling in skin development, homeostasis and disease, hair follicle development, and skin and hair follicle adult stem cells biology, althoughtly homozygous PLCD1-deficient mice don’t display completely hairless phenotype as expected.

## Additional file

**Additional file 1: Table S1.** Target loci of mouse PLCD1 gene. **Table S2.** Generation of PLCD1-modified mice (F0). **Table S3.** Primers for qRT-PCR analysis of PLCD1and the related gene expression. **Table S4.** List of putative off-target sites (OTSs). **Table S5.** Primer pairs for PCR amplification of OTSs.
