# Supplementary material for: Abnormalities of hair structure and skin histology derived from CRISPR/Cas9-based knockout of phospholipase C-delta 1 in mice
Source: J Transl Med. 2018 May 25;16:141. doi: 10.1186/s12967-018-1512-9 (PMC5968471; doi:10.1186/s12967-018-1512-9)
Supplement: Supplementary file 1 — Additional file 1: Table S1. Target loci of mouse PLCD1 gene. Table S2. Generation of PLCD1-modified mice (F0). Table S3. Primers for qRT-PCR analysis of PLCD1and the related gene expression. Table S4. List of putative off-target sites (OTSs). Table S5. Primer pairs for PCR amplification of OTSs. [file 12967_2018_1512_MOESM1_ESM.doc]

**Additional Tables**

**Additional Table S1** Target loci of mouse PLCD1 gene

| **sgRNA** | **Targeting loci** | **Target sequence（5’-3’）** | **PAM sequence** |
| --- | --- | --- | --- |
| sgRNA1 | m-PLCD1-Exon2 | CGTTCTCTACGCCACGAGC | TGG |
| sgRNA2 | m-PLCD1-Exon7 | CTTTTCTGCGACGTGCTC | AGG |
| sgRNA3 | m-PLCD1-Exon10 | GGTGCCGGAGCTCTCCGACA | TGG |

**Additional Table S2 Generation of PLCD1-modified mice (F0)**

| **No. of trial** | **Injected** | **Survived** | **Pups (ID)** | **No. of GMO** | **No. of Bi-allelic GMO** |
| --- | --- | --- | --- | --- | --- |
| 1 | 40 | 25 | 19, 20 | 0 | - |
| 2 | 87 | 78 | 1-6, 11-16, 21-25 | 7 | 3 |
| 3 | 64 | 53 | 31-39 | 4 | 1 |
| Total | 191 | 156 | 28(17.9%) | 11(42.3%) | 4(14.3%) |

**GMO: genetically modified organism**

**Additional Table S3 Primers for qRT-PCR analysis of PLCD1**

**and the related gene expression**

| **Gene** | **Forward primer (5’-3’)** | **Reverse primer (5’-3’)** |
| --- | --- | --- |
| PLCD1 | TGCCGAAGGTCAATAAGAA | TGGTAATAACTGCTGTCTGA |
| PLCB1 | GCAGAGTGTGCGTTCAAGAC | AATCGGCAATACTCGGCCAT |
| PLCG1 | CCCTTCTTGGAAACCAACGC | GGCTCCTCAATCTCTCGCAA |
| PLCE1 | TTCCGTGCTACCCTCCAAAG | AATTGGCCATAGAGGTGCCC |
| Krt1 | ACCAACGCAGAGAATGAG | GCAGAGAAGAAGTCAATGTC |
| Krt5 | TTAACAACCTCCGTAGACAG | CAGCATCCACATCCTTCTT |
| Krt13 | TCTCCATTGCTCCACACAGC | GGATGTCCTTTGGAGGGTAGC |
| Loricrin | GCAACGGAGACAACAGAG | GCGACTCAATGGCTTCTT |
| Involucrin | GAGAGCCATCCACACTTC | CCATATAGCATCCTGTATCCA |
| GAPDH | ACCTGCCAAGTATGATGAC | CTGTTGCTGTAGCCGTAT |

**Additional Table S4 List of putative off-target sites (OTSs)**

| **OTSs** | **Sequence** | **Mismatches** | **UCSC gene** | **locus** |
| --- | --- | --- | --- | --- |
| OTS1 | GGGTTCTCTTCGCCAGTAGCGAG | 4MMs[2:10:16:17] | NM_182999 | chr4:+49668960 |
| OTS2 | CCGTTCTCCACCCCAGGAGCAGG | 4MMs [1:9:12:16] | NM_009109 | chr7:-29867891 |
| OTS3 | GCGTTCTCTACACAAAGATCCGG | 4MMs[12:14:16:19] | NM_008480 | chr17:+68133424 |

**Additional Table S5 Primer pair**s for PCR amplification of OTSs

|  | **Forward primer (5’-3’)** | **Reverse primer (5’-3’)** |
| --- | --- | --- |
| OTS1 | CTGACCTGCCCTTGCTGTAA | TCCCTTCTCAGTGCCCAAAC |
| OTS2 | ACCCCTACCATGCCTCCTAC | TGAAACTTCAGGCGAGATTGT |
| OTS3 | ACCATGGAGGGACATTGCTG | CGCCATGACAGACTCGACTT |
